# Disparities in HIV and syphilis prevalence and risk factors between older male clients with and without steady sex partners in southwestern rural China

**DOI:** 10.1186/s12879-017-2367-z

**Published:** 2017-04-12

**Authors:** Li Chen, Jenny H. His, Xinghua Wu, Zhiyong Shen, Huaxiang Lu, Huanhuan Chen, Hui Huang, Heng Zhang, Yuhua Ruan, Yiming Shao, Zhenzhu Tang

**Affiliations:** 1 Guangxi Zhuang Autonomous Region Center for Disease Control and Prevention, No.18 Jinzhou Road, Nanning, Guangxi China; 2 State Key Laboratory of Infectious Disease Prevention and Control, National Center for AIDS/STD Control and Prevention, Chinese Center for Disease Control and Prevention, Collaborative Innovation Center for Diagnosis and Treatment of Infectious Diseases, Beijing, China

**Keywords:** HIV, Syphilis, Older male clients, FSWs, Risk behavior

## Abstract

**Background:**

Heterosexual intercourse accounted for 93% of reported HIV cases in Guangxi, and Guangxi had 10% of China’s total number of reported HIV cases. Older men are particularly vulnerable to STIs, for example, 46% of Guangxi’s HIV cases were men over 50 years of age. As this is an under-studied population in China, effective prevention and control policies have yet to be developed. Thus, the aim of this study was to use a large-scale cross-sectional survey to understand the demographic and behavior factors associated with HIV and syphilis infections among older male clients of female sex workers (FSWs) in a high epidemic area of rural Guangxi, China.

**Methods:**

A large-scale cross-sectional survey was conducted in 2012 among older male clients of FSWs in low-cost commercial sex venues. Questionnaire interviews were administered to collect sociodemographic and sexual behavior information. Blood samples were collected for HIV and syphilis infection tests.

**Results:**

Of the 3485 participants, 2509 (72.0%) clients had a steady sex partner and 976 (28.0%) clients had no steady sex partner. The overall prevalence of HIV and syphilis infection were 3.0% and 3.2%, respectively. Compared to those with a steady sex partner, clients with no steady partner had higher odds of HIV infection (AOR: 1.90, 95% CI: 1.27–2.86), syphilis infection (AOR: 1.53, 95% CI: 1.02–2.30), and having factors associated with HIV or syphilis infection, including non-commercial casual sex encounters in last month (AOR: 3.29, 95% CI: 2.42–4.46), >10 years of commercial sex history (AOR: 1.31, 95% CI: 1.12–1.53), >2 incidents of commercial sex in last month (AOR: 1.53, 95% CI: 1.19–1.96), and aphrodisiac use in last month (AOR: 1.40, 95% CI: 1.16–1.70). Clients with no steady partner had lower odds of having heterosexual intercourse (AOR: 0.66, 95% CI: 0.56–0.79), awareness and knowledge of HIV/AIDS (AOR: 0.75, 95% CI: 0.64–0.88), and having had HIV tests (AOR: 0.65, 95% CI: 0.44–0.98).

**Conclusion:**

Older male clients of low-cost commercial sex venues in rural southwestern China are at high risk for HIV and syphilis infection, especially those with no steady sex partner. Improved interventions are urgently needed for this neglected risk population.

## Background

In the 1960s, there was a government-led nationwide campaign, which was focused on the commercial sex trade, including a patriotic mass screening campaign, eradication of prostitution using severe and often punitive approaches, education and occupational training for prostitutes [1]. As a result, syphilis infections in China were virtually eliminated and other STIs were extremely uncommon. However, rapid social reform and economic development since 1978 have led to the re-flourishing of commercial sex activities across the country. As such, STI epidemics have become an increasingly important public health problem, including the new threat of HIV [2–5]. In its earlier decades in China, HIV was predominantly transmitted through blood routes. In 2007, 39% of reported HIV/AIDS cases were transmitted through heterosexual intercourse, while 29% were transmitted through injection drug use. In 2014, these proportions shifted to 66% and 6%, respectively [6, 7]. This transition, similar to those observed in other Asian countries, is likely due in part to the relatively strong effectiveness of interventions that target injection drug use [8, 9].

As in other Asian countries, the HIV epidemic evolved in China from a drug-driven epidemic in southern China in the 1990s to one fueled by sexual activity in the 2000s [5, 8, 9]. A particular area of interest for the rising sexual epidemic of HIV and STIs is the Guangxi Zhuang Autonomous Region in southwestern China. Located along major drug trafficking routes from the “Golden Triangle Region” and Yunnan to Guangdong and Hong Kong, Guangxi’s first cases of HIV infection were reported in 1996 by injection drug users (IDUs), IDUs accounted for more than two-third of all reported HIV cases in Guangxi until 2003, and injection drug use was the main mode of transmission before 2006 [10]. Since then, sexually transmitted HIV infections have constantly increased and reached 90% by 2012. In 2014 heterosexual intercourse accounted for 93% of reported HIV cases in Guangxi, and Guangxi had 10% of China’s total number of reported HIV cases [11]. Also, China’s population is steadily aging, and the elderly constitute a growing number of STI/HIV cases [9]. Older men are particularly vulnerable to STIs, as lack of knowledge of sexual health coupled with traditional concepts of sex and gender give rise to high-risk behaviors [12–17]. For example, 46% of Guangxi’s newly reported HIV cases in 2014 were men over 50 years of age [11]. Due to a dearth of information on HIV transmission patterns and risk factors in this new risk group, effective prevention and control policies have yet to be developed. Based on data from the HIV/AIDS case reporting system [10, 11] and sentinel surveillance data [16, 17] in Guangxi, we found that older male clients of female sex workers are at high risk of HIV infection. As well, a recent case-control study found that aphrodisiac use is a newly emerging risk behavior associated with HIV infection [18]. Many people over 50 years of age are still sexually active, and especially older men without a spouse may seek heterosexual activity with female sex workers (FSWs) or casual partners. Thus, the aim of this study was to use a large-scale cross-sectional survey to understand the demographic and behavior factors associated with HIV and syphilis infections among older male clients of female sex workers in a high epidemic area of rural Guangxi, China. In particular, we compared the risk and associated factors between male clients with and without a steady sex partner outside of their commercial sex activities.

## Methods

### The sampling method

This cross-sectional study was conducted in 2012 among older male clients of FSWs in low-cost commercial sex venues in 13 of Guangxi’s 14 prefectures (Liuzhou, Guilin, Guigang, Beihai, Binyang, Luzhai, Duan, Daxin, Lingshan, Pingnan, Quanzhou, Lingchuan, and Qintang). These survey sites were selected because of their high numbers of reported HIV cases and geographical representativeness. Approximately 250 participants were recruited from each site. The sex work venues in each survey site were systematically mapped out by experienced outreach staff, who then conducted community-based outreach to distribute study-related information to male clients and invite them to voluntarily participate in the study. Outreach workers also invited clients, FSWs, and brothel managers to refer other male clients to the study. Venues where the cost for a single sexual service is less than US $6 (1 RMB is roughly equivalent to 0.16 US dollar) are defined as low-cost venues, including inexpensive hotels, rooms rented by the hour, and outdoor settings. The study eligibility criteria were: heterosexual men only, over 50 years of age, self-reported to have had commercial sex with FSWs, and able and willing to provide informed consent. The definition of “older man” as over 50 years of age is based on a previous consensus in HIV/AIDS research [9, 12, 13, 18]. Subjects were excluded if they could not answer the sensitivity questionnaire, each eligible study participant completed a HIV/STI risk assessment interview, received HIV pre-test and risk reduction counseling, and had a blood specimen drawn for HIV and syphilis testing. Participants were informed of their test results and provided post-test counseling according to the results. Additional details of this study have been described in a case-control study which drew on this sample [18]. The study protocol and informed consent form were approved by the Institutional Review Board (IRB) of the Guangxi Center for Disease Control and Prevention. All study participants consented to take part in the study, e.g. completing questionnaires etc.

### Data collection

Each study participant was assigned a unique and confidential identity number. Survey data were collected using an interviewer-administered questionnaire. Sociodemographic and health characteristic variables included age, ethnicity, education, occupation, steady sex partner (defined as spouse or girlfriend with whom you have had sex for at least six months), self-reported health status, chronic disease (such as hypertension, diabetes, and metabolic arthritis at the time they were interviewed), and erectile function. Sexual or other behavioral variables included heterosexual intercourse, non-commercial casual sex partners, average cost per sexual transaction, history of commercial sex, number of commercial sexual intercourses, condom use, aphrodisiac use in the last month, awareness of HIV/AIDS and of available HIV voluntary counseling & testing (VCT) services, and HIV testing history. A standard questionnaire on HIV/AIDS knowledge developed by the Chinese Center for Disease Control and Prevention (CDC) was administered to the study subjects, and those who answered six of eight questions correctly were classified as “having awareness of HIV/AIDS”. Study interviewers were male staff from the local CDCs who could communicate with the participants using both standard Mandarin Chinese and the local dialects. Interviewers could help the participants to recall events and frequencies in a systematic way when necessary.

### Laboratory tests

Blood specimens were tested for both HIV and syphilis antibodies. The HIV antibody was tested by enzyme-linked immunosorbent assay (ELISA; Wantai Biological Production Company, Beijing, China) and was confirmed by an HIV-1/2 Western Blot immune assay (HIV Blot 2.2 WB, Genelabs Diagnostics, Singapore). The syphilis antibody was tested by the toluidine red unheated serum test (TRUST; Wantai Biological Production Company, Beijing, China) and confirmed by the Treponema pallidum particle agglutination assays (TP-PA) using the SERODIA-TPPA kit (FUJIREBIO INC., Tokyo, Japan).

### Data analysis

Questionnaires and laboratory testing data were double entered into EpiData software (EpiData version 3.1, The EpiData Association Odense, Denmark). After validation and cleaning, the data were then transferred into a SAS database for analysis (SAS 9.1 for Windows; SAS Institute Inc., NC, USA). To compare crude differences between the older male clients who had no steady sex partner and those who had. Chi-square tests and t-tests were used for categorical and continuous variables, respectively. Simple logistic regression was used to assess differences in the odds of HIV infection and syphilis infection, and display various behavioral risk factors for infection, respectively, between clients without a steady sex partner and those with one. Multivariable logistic regression was used to evaluate the same while controlling for age, ethnicity, education level, occupation, self-reported health status, chronic disease, and erectile function.

## Results

Our cross-sectional study enrolled 3485 older male clients from low-cost commercial sex venues in Guangxi, China. Of these, 2509 (72.0%) clients had a steady sex partner and 976 (28.0%) clients had no steady sex partner. Among those who had a steady sex partner, for 2466 clients (98.0%) she was a spouse or wife. Table 1 compares the sociodemographic and health characteristics between clients who had no steady sex partner and who did, as well as crude *P*-values to assess the significance of differences. The clients’ ages ranged from 50 to 92 years old. Compared to participants who had a steady sex partner, those who had no steady sex partner were less likely to belong to the younger age group of 50–59 years (29.2% vs. 39.1%, *p* < 0.001), less likely to be of Han ethnicity (64.7% vs. 69.2%, *p* = 0.01), more likely to have fewer years of education (80.0% vs. 66.8%, *p* < 0.001), less likely to have reported a good health status (77.7% vs. 85.7%, *p* < 0.001), and more likely to have erectile dysfunction (56.8% vs. 45.4%, *p* < 0.001). No significant differences between the two client groups were found in the other study variables, including occupation and chronic disease status.

**Table 1:** Sociodemographic and health characteristics of older male clients in Guangxi, China who had a steady sex partner and those who had no steady sex partner in 2012

Variable	Older male clients who had a steady sex partner *N* (%)	Older male clients who had no steady sex partner *N* (%)	*P*
Total	2509	976	
Age (years)			
50–59	980 (39.1)	285 (29.2)	<0.001
60–69	1012 (40.3)	333 (34.1)	
70+	517 (20.6)	358 (36.7)	
Ethnicity			
Han	1736 (69.2)	631 (64.7)	0.01
Others	773 (30.8)	345 (35.3)	
Years of education			
≤ 6	1677 (66.8)	781 (80.0)	<0.001
> 6	832 (33.2)	195 (20.0)	
Occupation			
Farmer	1937 (77.2)	769 (78.8)	0.312
Non-farmer	572 (22.8)	207 (21.2)	
Self-reported health status			
Very good	2150 (85.7)	758 (77.7)	<0.001
Fair/Not sure	359 (14.3)	218 (22.3)	
Chronic disease			
No	921 (36.7)	403 (41.3)	0.12
Yes	1588 (63.3)	573 (58.7)	
Erectile dysfunction			
No	1139 (45.4)	554 (56.8)	<0.001
Yes	1370 (54.6)	422 (43.2)	

Table 2 compares the behavioral characteristics and HIV and syphilis infection rates between the two client groups, as well as crude *P*-values for significance. Compared to participants who had a steady sex partner, those who had no steady sex partner were less likely to have had heterosexual intercourses in last month (46.7% vs. 61.9%, *p* < 0.001), more likely to have had non-commercial casual sex partners in last month (9.5% vs. 3.9%, *p* < 0.001), more likely to have used lower-charge sexual transactions (82.6% vs. 78.9%, *p* = 0.015), more likely to have had a longer history of commercial sex (41.0% vs. 33.6%, *p* < 0.001), more likely to have had unprotected sex with FSWs in the past (89.9% vs. 84.7%, *p* < 0.001), more likely to have used aphrodisiacs in the last month (22.5% vs. 16.8%, *p* < 0.001), less likely to know about HIV/AIDS (58.9% vs. 70.0%, *p* < 0.001), less likely to know about VCT services (15.2% vs. 18.3%, *p* = 0.027), less likely to have received HIV tests (3.2% vs. 5.9%, *p* = 0.001), more likely to have a syphilis infection (4.2% vs. 2.8%, *p* = 0.033), and more likely to have an HIV infection (4.5% vs. 2.4%, *p* < 0.001). No significant differences between the two groups of clients were found in the other study variables, including having had more than two incidences of commercial sex in the last month, having had any commercial sex in the last month, and having had unprotected sex with FSWs in last month.

**Table 2:** Unadjusted effects of sex partner status among older male clients (having no steady sex partner vs. having a steady sex partner) on the odds of HIV and syphilis infection and behavioral risk factors in 2012

Variable	Older male clients who had a steady sex partner *N* (%)	Older male clients who had no steady sex partner *N* (%)	OR^a^ (95% CI)
Total	2509	976	
Syphilis infection			
No	2439 (97.2)	935 (95.8)	
Yes	70 (2.8)	41 (4.2)	1.53 (1.03–2.26)
HIV infection			
No	2450 (97.6)	932 (95.5)	
Yes	59 (2.4)	44 (4.5)	1.96 (1.32–2.92)
Had heterosexual intercourses in the last month			
No	955 (38.1)	520 (53.3)	
Yes	1554 (61.9)	456 (46.7)	0.54 (0.46–0.63)
Had non-commercial casual sex partners in the last month			
No	2412 (96.1)	883 (90.5)	
Yes	97 (3.9)	93 (9.5)	2.62 (1.95–3.52)
Average charge per sex act (US $)			
≤ 4.8US $	1980 (78.9)	806 (82.6)	
> 4.8US $	529 (21.1)	170 (17.4)	0.79 (0.65–0.96)
History of commercial sex (years)			
≤ 10	1667 (66.4)	576 (59.0)	
> 10	842 (33.6)	400 (41.0)	1.37 (1.18–1.60)
Number of commercial sexual intercourses in the last month			
≤ 2	2275 (90.7)	864 (88.5)	
> 2	234 (9.3)	112 (11.5)	1.26 (0.99–1.60)
Had commercial sex in the last month			
No	924 (36.8)	392 (40.2)	
Yes	1585 (63.2)	584 (59.8)	0.87 (0.75–1.01)
Had unprotected sex with FSWs in the last month			
No	1221 (48.7)	469 (48.1)	
Yes	1288 (51.3)	507 (51.9)	1.02 (0.88–1.19)
Had unprotected sex with FSWs in the past			
No	383 (15.3)	99 (10.1)	
Yes	2126 (84.7)	877 (89.9)	1.60 (1.26–2.02)
Used aphrodisiacs in the last month			
No	2088 (83.2)	756 (77.5)	
Yes	421 (16.8)	220 (22.5)	1.44 (1.20–1.73)
Awareness of HIV/AIDS			
No	753 (30.0)	401 (41.1)	
Yes	1756 (70.0)	575 (58.9)	0.61 (0.53–0.72)
Awareness of VCT			
No	2049 (81.7)	828 (84.8)	
Yes	460 (18.3)	148 (15.2)	0.80 (0.65–0.97)
Received HIV testing			
No	2361 (94.1)	945 (96.8)	
Yes	148 (5.9)	31 (3.2)	0.52 (0.35–0.78)

Notes: ^a^ *OR* odds ratio, *CI* confidence interval. Univariate regression was used to calculate the odds of HIV and syphilis infection and other behavioral risk factors for infection, associated with the dependent variable was sex partner status

Table 3 presents the unadjusted and adjusted effects of steady sex partner status on the odds of HIV and syphilis infection and associated risk behaviors among the male clients. Adjusted odds ratios (AOR) were calculated by adjusting for major socio-demographic and health statuses, including age, ethnicity, years of education, occupation, self-reported health status, chronic disease, and erectile dysfunction. After adjustments, older male clients who had no steady sex partner had 34% lower odds of having had heterosexual intercourses in last month (AOR: 0.66, 95% CI: 0.56–0.79), over three times the odds of having had non-commercial casual sex partners in the last month (AOR: 3.29, 95% CI: 2.42–4.46), 31% higher odds of having a history of commercial sex >10 years (AOR: 1.31, 95% CI: 1.12–1.53), 53% higher odds of having had more than two incidences of commercial sex in the last month (AOR: 1.53, 95% CI: 1.19–1.96), 40% higher odds of aphrodisiac use in the last month (AOR: 1.40, 95% CI: 1.16–1.70), 25% lower odds of having awareness of HIV/AIDS (AOR: 0.75, 95% CI: 0.64–0.88), 35% lower odds of having received HIV tests (AOR: 0.65, 95% CI: 0.44–0.98), 53% higher odds of having a syphilis infection (AOR: 1.53, 95% CI: 1.02–2.30), and 90% higher odds of having an HIV infection (AOR: 1.90, 95% CI: 1.27–2.86). No significant differences between the two groups were found in the other study variables, including average charge per sex act, having had commercial sex in the last month, having had unprotected sex with FSWs in last month, and awareness of VCT. HIV and syphilis prevalence among the age groups of 50–59 years, 60–69 years, and ≥70 years were 3.1%, 2.8%, and 4.0% (*P* > 0.05), and 2.6%, 2.9%, and 3.5% (*P* > 0.05), respectively.

**Table 3:** Adjusted effects of sex partner status among older male clients (having no steady sex partner vs. having a steady sex partner) on the odds of HIV and syphilis infection and behavioral risk factors in 2012

Variable	AOR^a^ (95% CI)
Syphilis (+)	1.53 (1.02–2.30)
HIV (+)	1.90 (1.27–2.86)
Had heterosexual intercourse in the last month	0.66 (0.56–0.79)
Had non-commercial casual sex partners in the last month	3.29 (2.42–4.46)
Average charge per sex act (US $) >4.8US $	0.98 (0.80–1.20)
History of commercial sex (years) >10 years	1.31 (1.12–1.53)
Number of commercial sex acts in the last month >2	1.53 (1.19–1.96)
Commercial sex in the last month	1.05 (0.89–1.24)
Had unprotected sex with FSWs in the last month	1.12 (0.96–1.31)
Had unprotected sex with FSWs in the past	1.27 (0.99–1.62)
Used aphrodisiacs in the last month	1.40 (1.16–1.70)
Awareness of HIV/AIDS	0.75 (0.64–0.88)
Awareness of VCT	0.98 (0.79–1.21)
Received HIV testing	0.65 (0.44–0.98)

Notes: ^a^ AOR, adjusted odds ratio. Multivariable regression was used to calculate the odds of infection and having other behavioral risk factors, adjusted by age, ethnicity, years of education, occupation, self-reported health status, chronic disease, and erectile dysfunction. The dependent variable for each model was sex partner status

## Discussion

In this study, we found a high prevalence of HIV infection among older male clients in low-cost commercial sex venues in Guangxi, southwestern China, including those who had no steady sex partner (4.5%) and those who had a steady sex partner (2.4%). These rates are much higher than the overall prevalence estimate of HIV in China (0.68%) [19]. Previous studies reported an HIV prevalence higher than 3% among male clients of FSWs in some areas in China. However, those rates may be partially attributable to local high injection drug use [20–23]. Also, HIV prevalence among high, middle and low-cost FSWs in Guangxi was reported to be 0.5%, 0.5%, and 1.9%, while syphilis prevalence was 3.2%, 4.5%, and 10.5%, respectively [24]. Of note, low-cost FSWs were often found to be older than high- or middle-cost FSWs, and the HIV and syphilis prevalence among these FSWs were 1.8% and 10.7% in 2012 [24]. In contrast, the current prevention efforts for HIV and STIs in China are mainly focused on traditional high risk populations such as injection drug users, MSM, and FSWs, all of whom are predominantly young persons. Thus, our data and previous findings point to a serious need for new prevention efforts focused on older persons, such as older male clients patronizing middle-aged FSWs in low-cost commercial sex venues.

Our study also found that the odds of HIV and syphilis infection among older male clients who had no steady sex partner were much higher those of who had one. Although older male clients with a steady sex partner had more heterosexual intercourses in the last month, those with no steady sex partner engaged in more high risk behaviors, including higher odds of having non-commercial casual sex partners in the last month, a longer history of commercial sex, and more incidences of commercial sex in the last month. These risk behaviors in our survey are consistent with biological variables of HIV and syphilis infection. In order to address the HIV/AIDS epidemic, Chinese harm reduction programs have been implemented at the national and local levels since 2004, including education, HIV testing and counseling, condom promotion, methadone maintenance therapy, and needle exchange [25, 26]. These strategies have been shown to be an effective means of reducing unprotected sex with clients among FSWs and HIV infection among IDUs [27, 28]. However, there was no evidence from this study to indicate that harm reduction programs have lowered the odds of HIV infection and related risk behaviors among older male clients in low-cost commercial sex venues, especially those with no steady sex partner.

Since many older men remain significantly sexually active, older men without a spouse or steady sex partner may seek sex with FSWs or casual partners [29]. Our study showed that older male clients in Guangxi, China were not well informed of HIV/AIDS knowledge, VCT, and HIV testing, especially those who had no steady sex partner. Also, approximately half of all male clients (with and without a steady partner) have had unprotected sex with FSWs. It suggests that current health education and prevention programs have overlooked the intersection of HIV and older persons. In addition, this study found older male clients with no steady sex partner were more likely to use aphrodisiacs during commercial sex. Aphrodisiacs can improve sexual function, which may mask the clients’ health status and reduce their awareness of their own HIV/STI infection status and risk. Therefore, it can be viewed as a marker of a high-risk behavioral pattern for HIV transmission [18]. Many aphrodisiacs are illegally produced in China by informal, underground pharmacies or workshops, and urgent regulatory action from food and drug authorities is required.

Findings from the study should be interpreted in light of some limitations. First, participants were recruited using multiple approaches after the venues of sex work in each survey site were systematically mapped, and therefore, they were a convenient sample. Second, personally sensitive information regarding HIV and sexual behaviors was based on self-reporting, in which under-reporting may have biased the results due to social desirability. Third, due to social norm pressure, the potential study participants could not be recruited if they self-reported to be under 50 years of age or did not have heterosexual intercourse, which means that our sample might not be representative enough to make overall inferences of the population. Despite these limitations, our study provides scientific evidence on older male clients from low-cost commercial venues, a previously under-studied population in China, and shows that they are a high risk population, especially those with no steady sex partner. In China, Guangxi remains one of the most epidemic regions for HIV/AIDS, with significant numbers being older, male, rural, and having a low level of education. Heterosexual transmission has become the steady mode of transmission in Guangxi and the great majority of China, and this risk population may pose greater challenges for future control and prevention efforts. Combined with field epidemiological surveys in future studies, phylogenetic analysis should be used to interpret the source and social networks of HIV transmission in this high risk group [30].

## Conclusions

Older male clients of low-cost commercial sex venues in rural southwestern China are at high risk for HIV and syphilis infection, especially those with no steady sex partner. Improved interventions are urgently needed for this neglected risk population. Health authorities should develop new and evidence-based intervention approaches for such a significant and neglected risk population.

## Abbreviations

AIDS: Acquired immune deficiency syndrome
AOR: Adjusted odds ratios
China CDC: Chinese center for disease control and prevention
CI: Confidence interval
FSWs: Female sex workers
HIV: Human immunodeficiency virus
OR: Odds ratios
STI: Sexually transmitted infection
VCT: HIV voluntary counseling and testing
